# Scaling in vitro activity of CYP3A7 suggests human fetal livers do not clear retinoic acid entering from maternal circulation

**DOI:** 10.1038/s41598-019-40995-8

**Published:** 2019-03-15

**Authors:** Ariel R. Topletz, Guo Zhong, Nina Isoherranen

**Affiliations:** Department of Pharmaceutics, University of Washington School of Pharmacy, Box 357630, Seattle, WA 98195 USA

## Abstract

All-*trans*-retinoic acid (*at*RA), the active metabolite of vitamin A, is a critical signaling molecule during embryonic and fetal development and is necessary for maternal health. Fetal exposure to endogenous *at*RA is tightly regulated during gestation in a tissue specific manner and maternal exposure to exogenous retinoids during pregnancy is teratogenic. The clearance of *at*RA is primarily mediated by the cytochrome P450 (CYP) 26 enzymes, which play an essential role in controlling retinoid gradients during organogenesis. We hypothesized that CYP26 enzymes in the human fetal liver also function as a protective barrier to prevent maternal *at*RA reaching fetal circulation. Using human fetal liver tissue, we found that the mRNA of CYP26A1 and CYP26B1 enzymes is expressed in the human fetal liver. However, based on inhibition studies, metabolite profiles and correlation of *at*RA metabolism with testosterone hydroxylation, clearance of *at*RA in the fetal livers was mediated by CYP3A7. Based on *in vitro*-to-*in vivo* scaling, *at*RA clearance in the fetal liver was quantitatively minimal, thus providing an insufficient maternal-fetal barrier for *at*RA exposure.

## Introduction

All-*trans*-retinoic acid (*at*RA) is the key active metabolite of vitamin A (retinol). *at*RA is a critical morphogen and a signaling molecule during embryonic and fetal development. Carefully controlled concentration gradients of *at*RA in the developing embryo are needed to mediate organogenesis and other developmental processes^1–3^. *at*RA and its isomer 13-*cis*RA are well characterized teratogens in humans and in model species, and dosing of pregnant mothers with these retinoids results in severe fetal malformations^4–6^. Maternal deficiency of vitamin A is also associated with fetal malformations^7,8^. It is generally believed that *at*RA concentrations in specific cells in the developing embryo are controlled by gestational age, cell-type specific expression of *at*RA-synthesizing aldehyde dehydrogenase 1A (ALDH1A) enzymes and *at*RA metabolizing cytochrome P450 (CYP) family 26 (CYP26) enzymes, resulting in the necessary *at*RA gradients that regulate differentiation, proliferation and apoptosis of cells within the embryo^3,9^. The fetus appears to be dependent on a maternal supply of retinol, as evidenced by placental expression of Stra6, the uptake transporter for retinol^10^, and the severe birth defects observed in individuals carrying Stra6 mutations^11^. Similarly, studies in several model species have shown that disruption of *at*RA synthesis or metabolism via knocking out ALDH1A2, ALDH1A3, CYP26A1 or CYP26B1 in the embryo/fetus is detrimental, resulting in severe malformations and unviable offspring^9,12^. As such, it is expected that xenobiotics or environmental and genetic factors that alter the activity of these enzymes will result in teratogenicity due to too low or excessive exposure to *at*RA in the fetus. However, at present the processes that prevent fetal exposure to maternal circulating *at*RA to allow the fetus to independently regulate its retinoid gradients are not known.

In the adult human liver, *at*RA has been shown to be mainly metabolized by CYP enzymes CYP26A1, CYP26B1, CYP3A and CYP2C8^13,14^. While CYP3A and CYP2C8 mainly hydroxylate *at*RA at the C4-position forming 4-OH-RA, CYP26 enzymes oxidize *at*RA at several sites resulting in the formation of 4-OH-RA as well as 16- and 18-OH-RA^15,16^. The 4-OH-RA is then sequentially oxidized to 4-oxo-RA by an unknown alcohol dehydrogenase^17^. In adult human liver, CYP26A1 has been shown to be the main *at*RA hydroxylase likely regulating *at*RA clearance^13^. CYP26A1 and CYP26B1 have also been shown to play an important role in regulating *at*RA homeostasis during mouse development, and these enzymes are expressed during mouse development in a tissue and gestational age dependent manner^9,18,19^. However, information regarding CYP26 activity or expression in human fetal organs is limited, and it is unknown whether the same CYP26 enzymes that appear predominant in metabolizing *at*RA in adult human liver also regulate *at*RA clearance in the fetal liver. In fact, clear dichotomy of expression of CYP enzymes between fetal and adult livers has been shown for the CYP3A family of enzymes^20–22^. While CYP3A4 and CYP3A5 are the main CYP3A enzymes in adult human liver, they are not well expressed in the fetal liver. Instead, CYP3A7 is the main human fetal liver CYP3A isoform^20–22^. CYP3A7 also metabolizes *at*RA^13,23^ and before the identification of CYP26 enzymes, CYP3A7 was suggested as the main fetal liver *at*RA hydroxylase limiting fetal exposure to *at*RA^23^. Although CYP26 mRNA has been previously detected in human fetal liver tissues^24–26^, the quantitative importance of the CYP26 enzymes and CYP3A7 in modulating maternal-fetal transfer of *at*RA and fetal *at*RA clearance is not known. Based on the existing data that show the importance of CYP26 enzymes in *at*RA metabolism in adult liver and the importance of tight regulation of retinoid gradients in the developing fetus, we hypothesized that CYP26A1 plays an important role in fetal *at*RA clearance, and that the fetal liver limits maternal-fetal transfer of *at*RA. The aims of this study were to determine whether CYP26 enzymes are important in *at*RA clearance in human fetal liver, whether CYP3A7 contributes to *at*RA clearance in human fetal liver and to determine the efficiency of the fetal liver in eliminating *at*RA that passes to the fetus from maternal circulation.

## Results

### Detection of CYP26 mRNA in human fetal livers

**Figure 1:**
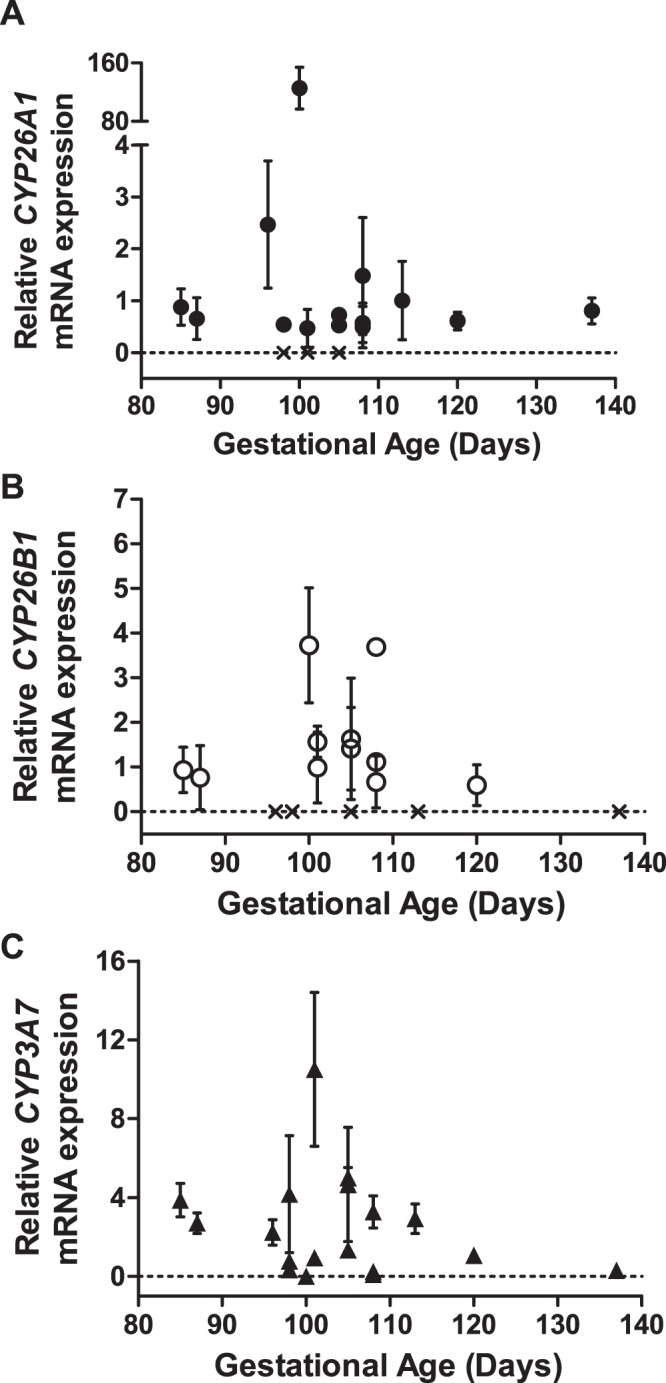
Expression of *CYP26A1* (**A**, closed circles), *CYP26B1* (**B**, open circles) and *CYP3A7* (**C**, closed triangles) mRNA in human fetal livers. The mRNA expression in each fetal liver was measured as duplicates and repeated in three separate days. The data shown is the mean ± S.D. from the three different experiments. The symbol “X” indicates a sample with no detectable mRNA expression. The mRNA expression of *CYP26A1* and *CYP26B1* was undetectable in 4 and 7 of all 18 fetal livers, respectively, while *CYP3A7* mRNA expression was detected in all fetal livers.

The mRNA expression of *CYP26A1*, *CYP26B1* and *CYP26C1* together with *CYP3A7* was measured in fetal livers from 18 individual donors (Fig. 1) and in five control adult human livers (data not shown). *CYP26A1* and *CYP26B1* mRNAs were detected and quantified in 14 and 11 of the 18 fetal livers, respectively, while *CYP26C1* mRNA was not detected in any of the 18 fetal livers. Considerable inter-individual variability, up to 125-fold, was observed in the expression of *CYP26A1* in the fetal livers while the expression of *CYP26B1* was less variable (Fig. 1). Two of the fetal livers had no detectable expression of any *CYP26* mRNAs and in both of these livers robust *CYP3A7* mRNA expression was detected. In fact, *CYP3A7* expression was relatively high (C_t_ values 26–34) in 17 of the 18 fetal livers. One fetal liver had very low *CYP3A7* expression (C_t_ value 38) and this liver showed the highest *CYP26A1* expression among all 18 fetal livers. The overall C_t_ values of *CYP26A1* were higher in the fetal livers (one with C_t_ value 32 and others 37–38) than in the adult livers analyzed (C_t_ 27–36) while *CYP26B1* C_t_ values were generally lower in the fetal livers (C_t_ 36–39) than adult livers (*CYP26B1* was only detectable in one adult liver). Due to differences in housekeeping gene expression between adult and fetal livers, no quantitative comparisons were made between fetal and adult liver mRNA expression. *CYP26C1* mRNA was not detected in any of the adult human livers while *CYP3A7* mRNA was detected in 3 of the 5 adult livers (C_t_ values 30–37).

### *at*RA metabolism in human fetal livers

**Figure 2:**
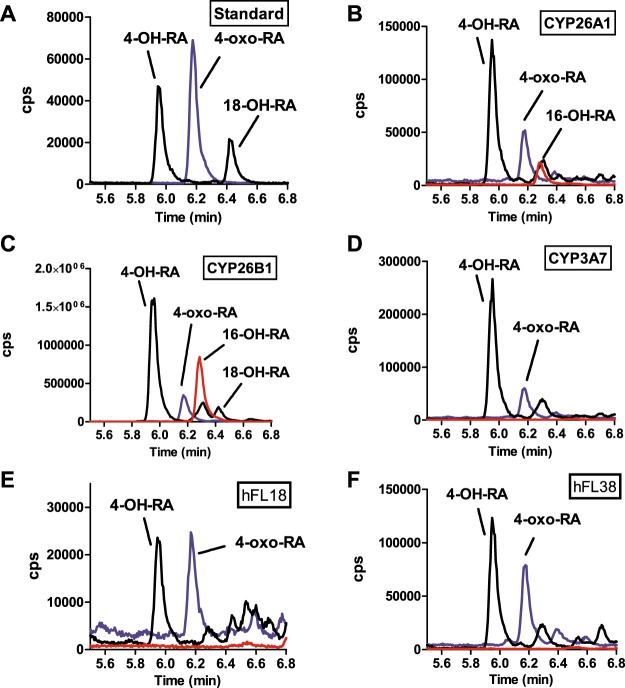
Identification of the metabolites formed from *at*RA by recombinant CYP26A1, CYP26B1, CYP3A7 and by human fetal livers. Panels A–D show representative chromatograms of metabolite standards (**A**) and incubations with recombinant CYP26A1 (**B**) CYP26B1 (**C**) and CYP3A7 (**D**). Panels E and F show the metabolite formation in S9 fractions from two representative human fetal livers (hFL). The individual donors are indicated with the sample number (18 and 38 listed in Table 1). The incubations were conducted as described in materials and methods. The *m/z* transitions of 315 > 253 Da (4-OH-RA and 18-OH-RA; black line), 313 > 269 Da (4-oxo-RA; blue line) and 315 > 241 Da (16-OH-RA; red line) were monitored by LC-MS/MS and the observed peaks are labeled in each panel.

To explore whether the metabolites formed from *at*RA in fetal liver resembled either the metabolite profile observed from *at*RA with recombinant CYP3A7 or CYP26 enzymes, the metabolites formed from *at*RA by CYP3A7, CYP26A1 and CYP26B1 and by fetal liver S9 fractions were characterized (Fig. 2). Consistent with previous data, recombinant CYP26A1 and CYP26B1 hydroxylated *at*RA at multiple sites, while CYP3A7 only formed 4-OH-*at*RA and 4-oxo-*at*RA. The metabolite profile in human fetal livers was similar to that observed with CYP3A7. Of the *at*RA metabolites, only the formation of 4-OH-RA and its metabolite 4-oxo-RA was observed (Fig. 2). No formation of the CYP26 specific metabolite 16-OH-RA was observed in fetal livers. The fetal liver metabolite profile corresponded to that observed with CYP3A7 and suggests lack of significant CYP26 contribution to *at*RA metabolism in fetal liver.

**Figure 3:**
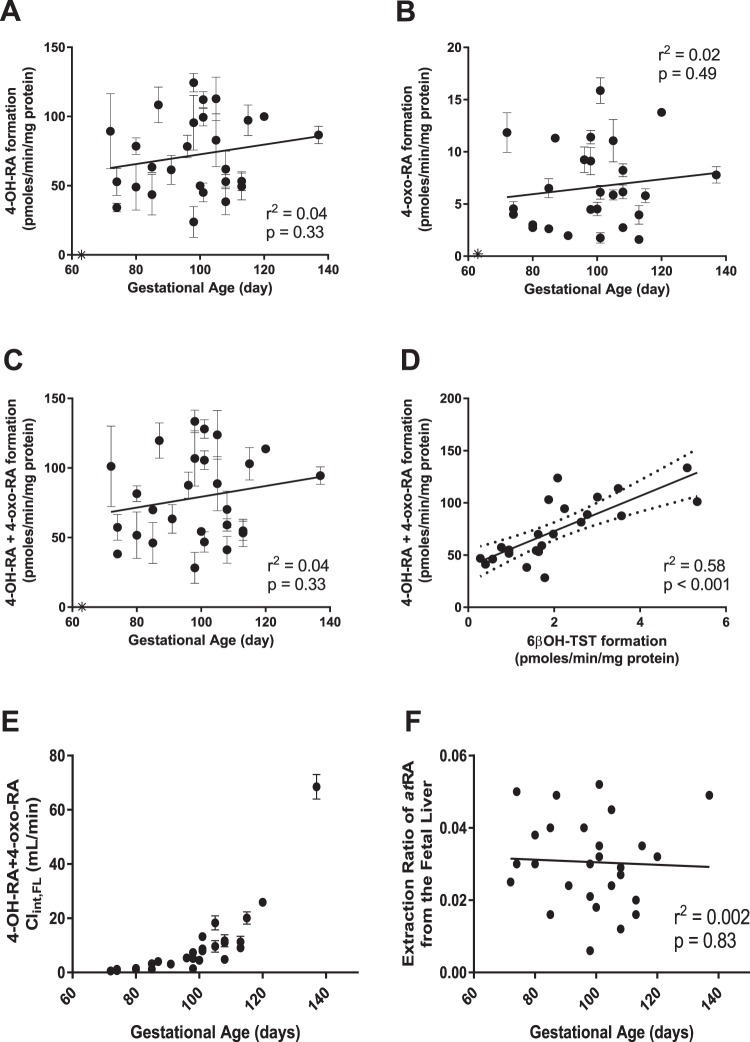
Quantitative analysis of *at*RA and testosterone (TST) metabolism in human fetal livers. Formation of 4-OH-RA (**A**), 4-oxo-RA (**C**) and the sum of 4-OH-RA and 4-oxo-RA (**E**) in individual human fetal livers from different gestational ages is shown. The insets list the correlation analysis between gestational age and product formation velocity. Panel D shows the correlation between *at*RA oxidation and 6βOH-TST formation in individual human fetal livers. Panel E shows the intrinsic clearance of *at*RA oxidation in the fetal livers (Cl_int,FL_) based on whole organ scaling from the S9 fraction data as described in the methods section. The calculated extraction ratio of *at*RA at different gestational ages is shown in panel F for individual human fetal livers.

The metabolism of *at*RA was quantified in 27 individual fetal livers from gestational ages between 72 and 137 days (Table 1, Fig. 3). The metabolite formation velocity ranged from 24 ± 11 to 124 ± 7 pmol/min/mg protein for 4-OH-RA, from 1.6 ± 0.1 to 16 ± 1 pmol/min/mg protein for 4-oxo-RA and from 28 ± 11 to 134 ± 8 pmol/min/mg protein for the sum of 4-OH-RA and 4-oxo-RA. There was no correlation between gestational age and the formation of 4-OH-RA (r^2^ = 0.04, *p* = 0.33), 4-oxo-RA (r^2^ = 0.02, *p* = 0.49), or their sum (r^2^ = 0.04, *p* = 0.33), and the metabolite formation velocity did not differ between weeks of gestation (Fig. 3, Table 2). There were no significant differences in metabolite formation between fetal sexes or maternal races (Table 2).

**Table 1:** Donor characteristics and fetal liver physiological parameters used.

Fetal Liver code	Gestational Age (Day)	Sex Of fetus	Race	Yield of S9 protein as mg/g fetal liver	Fetal liver weight (g)	Umbilical vein flow (mL/min)
FL 30	72	M	n/a	27	0.4	0.45
FL 20	74	M	n/a	70	0.5	0.45
FL 21	74	n/a	Asian	79	0.5	0.45
FL 36	80	n/a	Caucasian	43	0.9	0.78
FL 39	80	F	Asian/Hispanic	53	0.9	0.78
FL 24	85	n/a	Asian	75	1.2	1.6
FL 37	85	M	n/a	43	1.2	1.6
FL 14	87	F	n/a	47	1.4	1.6
FL 12	91	F	Asian	52	1.9	2.6
FL 31	96	M	Caucasian	46	2.6	2.6
FL 13	98	M	n/a	32	3.0	4.8
FL 27	98	F	Caucasian	36	3.0	4.8
FL 18	98	n/a	Asian	33	3.0	4.8
FL 34	100	M	n/a	48	3.4	4.8
FL 40	101	M	Caucasian	68	3.7	4.8
FL 33	101	M	n/a	93	3.7	4.8
FL 15	101	n/a	n/a	37	3.7	4.8
FL 23	105	M	Alaskan	46	4.7	7.8
FL 38	105	F	Caucasian	63	4.7	7.8
FL 19	108	F	Hispanic	60	5.6	7.8
FL 28	108	F	Asian	67	5.6	7.8
FL 29	108	F	n/a	42	5.6	7.8
FL 26	113	n/a	n/a	44	7.4	11.1
FL 25	113	M	n/a	57	7.4	11.1
FL 17	115	n/a	n/a	47	8.3	11.1
FL 16	120	M	Caucasian	42	10.7	15.6
FL 22	137	M	Alaskan	62	23.2	26.8

Each fetal liver (FL) is identified with a numerical sample code. The fetal liver weights listed are based on reported data for gestational days 72–137^40,41^. The unbilical vein blood flow was calculated based on the reported value of 111.7 mL/min/kg fetal weight^42^.

**Table 2:** Descriptive analysis of *at*RA oxidation in the different groups of fetal livers included in the analysis.

	4-OH-RA formation (pmol/min/mg protein)	4-oxo-RA formation (pmol/min/mg protein)	4-OH-RA+ 4-oxo-RA formation (pmol/min/mg protein)	4-oxo-RA/4-OH-RA (ratio)
gestational week 10–12 (n = 5)	61 ± 23	5.2 ± 3.8	66 ± 25	0.09 ± 0.04
gestational week 12–14 (n = 8)	75 ± 34	7.1 ± 3.7	82 ± 37	0.10 ± 0.05
gestational week 14–16 (n = 9)	73 ± 30	6.9 ± 4.3	80 ± 33	0.09 ± 0.03
gestational week 16–20 (n = 5)	77 ± 24	6.6 ± 4.6	84 ± 28	0.08 ± 0.04
Female Fetus (n = 8)	80 ± 25	7.5 ± 3.6	87 ± 28	0.09 ± 0.03
Male Fetus (n = 11)	75 ± 27	7.4 ± 4.7	83 ± 31	0.10 ± 0.05
Alaskan (n = 2)	106 ± 9	14.8 ± 1.5	121 ± 10	0.14 ± 0.00
Asian (n = 6)	76 ± 35	6.4 ± 3.6	82 ± 38	0.09 ± 0.04
Caucasian (n = 6)	73 ± 21	5.7 ± 3.1	79 ± 24	0.08 ± 0.03

There were no significant differences in *at*RA oxidation rates between gestational age groups, sex, or race.

### Identification of CYPs that metabolize *at*RA in human fetal livers

**Figure 4:**
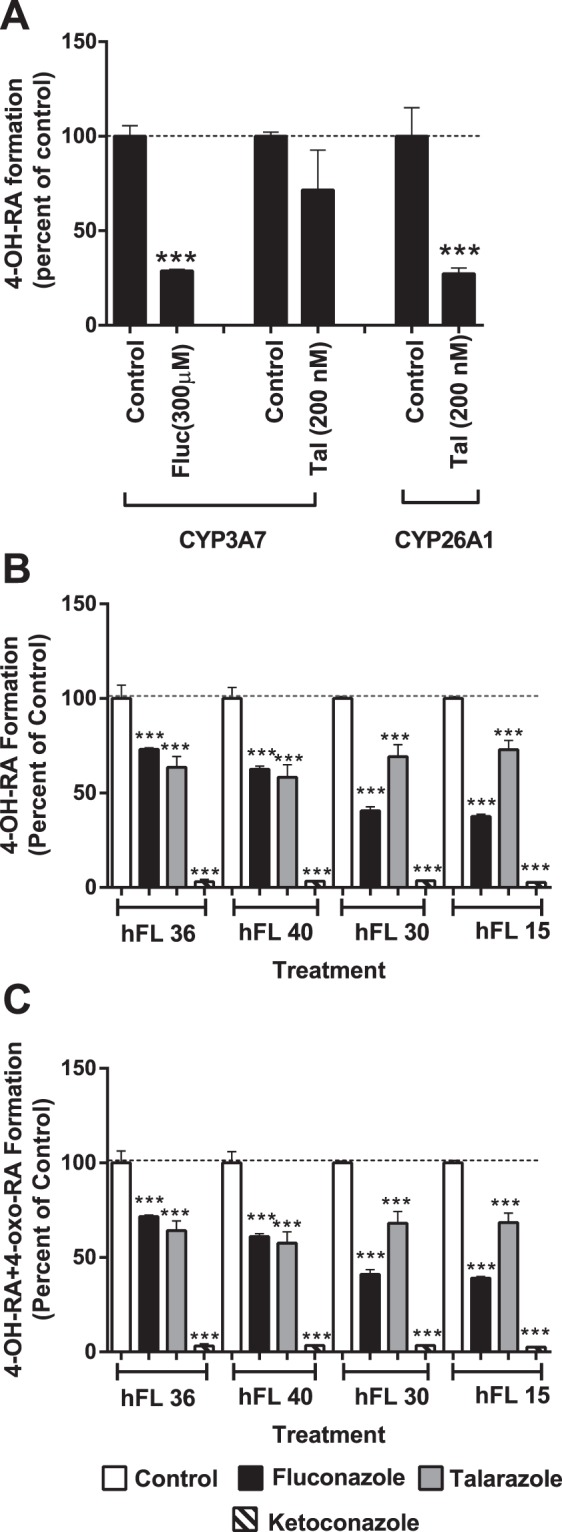
Inhibition of atRA metabolism by specific CYP inhibitors. Panel A shows the inhibition of 4-OH-RA formation by CYP3A7 inhibitor fluconazole (fluc, 300 μM) and the CYP26 inhibitor talarozole (tal, 200 nM). atRA concentrations were 10 μM and 500 nM in the CYP3A7 and CYP26 incubations. Panels B and C show the percentage inhibition of 4-OH-RA (**B**) and 4-OH-RA + 4-oxo-RA (**C**) formation in four representative human fetal livers (hFL, numbers correspond to sample identifiers) by fluconazole (black bars), talarozole (grey bars), and ketoconazole (striped bars) in comparison to vehicle controls (white bars). The stars (***) indicate a significant difference between the incubations with the indicated inhibitor and vehicle control. The numbers indicate the donor codes for the fetal liver donors.

To quantify which CYP enzymes contribute to *at*RA clearance in fetal liver, selective inhibitors of CYP3A7 and CYP26 were used. Fluconazole has been previously shown not to inhibit CYP26^16^. The inhibition of CYP3A7 mediated 4-OH-*at*RA formation by fluconazole (300 μM) was confirmed using recombinant CYP3A7. Inhibition of CYP26A1 by talarozole (200 nM) was reproduced with recombinant CYP26A1 (Fig. 4). The specificity of talarozole (200 nM) towards CYP26 was confirmed using recombinant CYP3A7. Talarozole did not significantly inhibit CYP3A7 mediated 4-OH-RA formation (Fig. 4). Together these data show that fluconazole and talarozole can be used as specific inhibitors of CYP3A7 and CYP26 respectively. In human fetal liver S9 fractions, *at*RA metabolite formation was inhibited 30–60% by fluconazole and 30–40% by talarazole (Fig. 4), suggesting that both CYP26 enzymes and CYP3A7 contribute to *at*RA clearance in human fetal liver. Ketoconazole inhibits both CYP26 enzymes and CYP3A7^16^. Consistent with this inhibition profile, ketoconazole caused a >95% decrease in *at*RA metabolite formation. However, these inhibition experiments do not unequivocally define which CYP enzyme is predominant in *at*RA clearance in human fetal liver. Therefore, to further define the importance of CYP3A7 in *at*RA clearance in fetal liver, correlation analysis between *at*RA metabolite formation and the formation of the CYP3A7 specific testosterone metabolite 6βOH-testosterone was conducted (Fig. 3). *at*RA oxidation (the sum of 4-OH-RA and 4-oxoRA formation) and 6βOH-testosterone formation from testosterone correlated significantly in the fetal livers tested (r^2^ = 0.58, *p* < 0.05) suggesting that CYP3A7 plays a major role in *at*RA metabolism in human fetal liver.

### Prediction of *at*RA clearance in the human fetal liver

To define the quantitative role of the fetal liver in *at*RA clearance and in potentially serving as a barrier to maternal-fetal retinoid transfer, the overall organ clearance and extraction ratio of *at*RA metabolism was calculated for the fetal liver for the gestational ages studied. The overall intrinsic clearance of *at*RA by the whole fetal liver (Cl_intFL_) increased with gestational age due to the growth of the fetal liver. The intrinsic clearance per mg S9 protein was independent of gestational age (Fig. 3). The predicted fraction of *at*RA removed from fetal circulation by the fetal liver (extraction ratio) was very low, ranging from 0.01 to 0.05 (Fig. 3F). This suggests minimal extraction of maternal *at*RA by the fetal liver.

## Discussion

*at*RA is a key developmental morphogen, and distinct concentration gradients of *at*RA within the developing embryo and fetus are crucial for regulation of cellular differentiation^1,2,7^. Embryonic development requires gestational age and tissue-type specific regulation of *at*RA concentrations^2,3,9^. Based on this, we hypothesized that a barrier, such as the fetal liver or the placenta, exists between the mother and the fetus to prevent maternal endogenous *at*RA passing to the fetus and enabling autonomous regulation of fetal *at*RA concentrations. Previous studies have shown CYP26 mRNA expression in fetal liver^24–26^. Hence, we hypothesized that the CYP26 enzymes, which are generally believed to be the main human retinoic acid hydroxylases^14,27^, would constitute a maternal-fetal barrier for maternal *at*RA. Based on the mRNA analysis of individual fetal livers, however, CYP26 enzymes appeared relatively insignificant in the human fetal liver both in terms of observed mRNA expression and apparent activity. At the same time, *CYP3A7* mRNA was abundant in the fetal livers, in agreement with past studies^20,21^. The finding of low but detectable expression of *CYP26A1* mRNA in the human fetal livers is similar to prior findings. One study showed low to undetectable *CYP26A1* mRNA in the human fetal liver and relatively high *CYP26A1* mRNA in human fetal cephalic tissue^25^. A second study in a single donor showed weak detection in a single donor^26^. The detection of *CYP26B1* mRNA in a subset of the fetal livers is consistent with the prior detection of *CYP26B1* mRNA in a single donor of human fetal liver^26^. The mRNA expression of *CYP26A1* and *CYP26B1* observed in the adult liver in this study agrees with previous reports showing that CYP26A1 is the predominant CYP26 enzyme in adult liver and CYP26B1 is either undetectable or has very low expression^13,15,26,28^. In contrast to the previous single donor analysis however^26^, *CYP26C1* mRNA was not detected in adult or fetal livers. The detection of high *CYP26A1* mRNA in one fetal liver that had very low CYP3A7 expression is of particular interest. If CYP3A7 is predominantly responsible for *at*RA clearance in the fetal liver, low expression or lack of this CYP would result in increased *at*RA concentrations in fetal liver. These increased *at*RA concentrations should in turn induce the expression of CYP26A1 in the fetal liver leading to the observed mRNA expression pattern. Although this observation is from a single donor, it is consistent with the prevailing notion that CYP26A1 expression is responsive to *at*RA concentrations and its expression is induced by increased *at*RA concentrations^17,28^.

Despite the detection of CYP26 isoform mRNA in human fetal livers, all the data presented here (the metabolite profiles, CYP inhibition data and correlation of 4-OH-RA formation with 6βOH-TST formation) support the role of CYP3A7 as the main human fetal liver *at*RA hydroxylase with only a minor contribution of CYP26 enzymes. This was surprising, as in adult human liver CYP26A1 was previously found to be the main *at*RA hydroxylase despite the activity of CYP3A4 and CYP3A5 as *at*RA hydroxylases^13^. Yet, even in the adult human liver, CYP3A4 and CYP3A5 expression did significantly correlate with *at*RA hydroxylation activity. In particular in livers with low CYP26A1 expression, CYP3A enzymes were likely to be significant *at*RA hydroxylases^13^. Although CYP3A7 constitutes 30–85% of the fetal liver CYPs^29^ we expected that the >1,000 fold higher intrinsic clearance of *at*RA by the CYP26 enzymes in comparison to CYP3A7^13,15,30^ would translate to an important contribution of CYP26s to fetal liver *at*RA clearance, even if their expression was much lower than CYP3A7. However, the data collected does not support this hypothesis and we conclude that CYP3A7 is the main human fetal liver *at*RA hydroxylase. This finding is likely to translate to other RA isomers, 13-*cis*RA and 9-*cis*RA as well, as they have similar clearance profiles by CYP26s as *at*RA^30^ and are metabolized by CYP3A7^23^. The lack of change in *at*RA and testosterone oxidation activity with gestational age observed in this study is consistent with previous reports that have shown relatively stable CYP3A7 expression across gestational ages^20,21^. The data is, however, discrepant with the study that showed higher *at*RA hydroxylation rates in three fetal livers from gestational days 96–109 when compared to livers from days 54–89 of gestation^23^. The previously observed change with gestational age is likely due to the small sample size that did not completely capture inter-individual variability in *at*RA clearance.

This is the first study to scale the observed microsomal or S9 protein activity to the entire fetal liver. This scaling shows that the metabolic capacity of the fetal liver towards *at*RA is unlikely to contribute significantly to fetal *at*RA clearance, and thus this metabolism is not quantitatively sufficient to protect the developing fetus. While the intrinsic clearance of *at*RA metabolism per mg of fetal liver protein did not change with gestational age, our analysis shows that when the growth of the liver and increase in umbilical blood flow is accounted for, the overall metabolic clearance of *at*RA increases significantly with gestational age. However, the extraction ratio is unchanged with gestational age, and the predicted extraction ratio (0.01–0.05) suggested that maximum 5% of maternal *at*RA could be extracted by fetal liver at any gestational age. The scaling methods used here, the analysis of the extraction ratio and the prediction of the overall fetal liver metabolic clearance are likely useful for future estimations of the role of the fetal liver in clearance of therapeutic drugs and toxins which are metabolized by fetal liver CYPs or glucuronidation enzymes.

Collectively, the data presented here are in agreement with the current consensus^2,3,9^ that fetal tissue exposure to *at*RA and tissue *at*RA concentrations and signaling are regulated at the individual organ level and not via a maternal-fetal barrier. It is also important to note that *at*RA signaling is most significant during early embryogenesis, a window of gestation that cannot be feasibly studied in human tissues. As such, concordance of the findings from this study with animal models will need to be established in studies in model organisms at gestational time periods after main sensitivity periods to *at*RA. During the early gestation, the placenta is likely to play a critical role in protecting the embryo, and *at*RA metabolism and transport in the human placenta requires further study. Interestingly in our preliminary studies, we did not observe any formation of *at*RA metabolites in human placental S9 preparations (data not shown) suggesting a lack of placental metabolic barrier. Further work is needed to define potential active transport mechanisms in the placenta that modulate species differences in fetal exposure to teratogenic retinoids^4–6^ and maternal-fetal transfer of retinoids during sensitive periods of development.

## Methods

### Chemicals and Reagents

*at*RA, ketoconazole, fluconazole, and talarazole were purchased from Sigma-Aldrich (St. Louis, MO). 4-oxo-RA-d_3_ and *at*RA-d_5_ were purchased from Toronto Research Chemicals (North York, Ontario). 4-OH-RA, 4-oxo-RA, and 18-OH-RA were synthesized as previously described^15,31^. Optima LC/MS-grade water, optima LC/MS-grade acetonitrile, ethanol and ethyl acetate were purchased from Fisher Scientific (Pittsburg, PA). Recombinant CYP3A7 supersomes coexpressed with P450 reductase were purchased from BD Gentest. Recombinant CYP26A1 and CYP26B1 were expressed in baculovirus infected insect cells and membrane fractions prepared via ultracentrifugation as previously described^15,32^.

### Collection of Human livers

The study was approved by the Institutional Review Board (IRB) at the University of Washington and the studies were conducted in accordance with the guidance of Office of Human Research Protections. Human fetal liver tissues (n = 27) ranging from reported gestational days 67 to 137 were collected by the Birth Defects Laboratory at the University of Washington and flash frozen upon collection using liquid nitrogen and stored at −80 °C until ready for use. The gestational days were based on self-reports and exact dates of conception are not known. Donated tissues were from elective abortions and all donor moms signed informed consent for donating the tissues. Tissues from fetuses whose mothers had known drug use were excluded from this study. Adult liver tissues (n = 5) were from de-identified donors from University of Washington human liver bank.

### Analysis of CYP26 mRNA expression

To explore the presence of CYP26 enzymes in human fetal liver, mRNA was extracted from 18 fetal livers and five adult human livers as previously described^28^. 50–70 mg of liver was homogenized in 2 ml Omni Hard Tissue Homogenizing tubes containing 1.4 mm ceramic beads and 1 ml of TRI reagent (Invitrogen; Grand Island, NY, USA). Homogenization was conducted using Omni Bead Ruptor 24 (Omni International; Kennesaw, GA, USA) and mRNA was extracted with TRI reagent according to the manufacturer’s recommendations. Total RNA was quantified using a Nanodrop 2000c Spectrophotometer (Thermo Fisher Scientific, Waltham, MA). cDNA was synthesized from 1 μg mRNA using TaqMan reverse transcription reagents (Applied Biosystems, Carlsbad, CA). The mRNAs of *CYP26A1*, *CYP26B1* and *CYP26C1* were quantified as target genes, *CYP3A7* as a control gene, and *β-actin*, *GAPDH* and *GUSB* were evaluated as housekeeping genes. Based on the variability in the gene expression, *β-actin* was chosen as the housekeeping gene. mRNA expression was quantified using StepOnePlus^TM^ q-RT-PCR (Applied Biosystems; Carlsbad, CA, USA). All samples were analyzed in duplicates and the q-RT-PCR repeated on three separate occasions. For samples that were undetected in one of the three experiments (5 samples for CYP26A1, 4 for CYP26B1 and 1 for CYP3A7) a C_t_ value of 40 was assigned to the undetected run and the mean of the three experiments was calculated. For samples that were undetected in two of the three experiments, samples were considered as target gene undetected (2 samples for CYP26A1 and 6 for CYP26B1). The relative abundance of *CYP26A1*, *CYP26B1*, *CYP26C1* and *CYP3A7* mRNA expression was analyzed by the ∆∆Ct method using *β-actin* as a housekeeping gene and the data are presented as a fold difference in comparison to the mean value for each gene. No comparisons for expression between genes and between adult and fetal livers were done. Human primer and probe pairs for CYP26A1 (Hs01075675_m1, FAM), CYP26B1 (Hs01011223_m1, FAM), CYP26C1 (Hs01595345_m1), CYP3A7 (Hs00426361_m1, FAM), ACTB (Hs01060665_g1, FAM), GUSB (Hs00939627_m1, FAM) and GAPDH (Hs02786624_g1, FAM) were obtained from Applied Biosystems (Carlsbad, CA, USA).

### Preparation of liver S9 fractions

Human fetal liver S9 fractions containing cytosol, cell membranes including microsomes, and small mitochondria and other small cell organelles were prepared to evaluate *at*RA metabolism in human fetal liver. For this 300 µL of 50 mM Potassium phosphate (KPi) buffer (pH 7.4) containing 250 mM sucrose, 1 mM EDTA and 1 mM PMSF was added to 0.1–0.3 g of fetal liver sample and the tissue was homogenized in 2 mL Omni Hard Tissue Homogenizing tubes containing 1.4 mm ceramic beads using a 2*20 sec cycles with an Omni Bead Ruptor 24 containing dry ice in acetone (Omni International, Kennesaw, GA). The homogenates were then centrifuged at 9,000 g for 20 min to pellet cell nuclei, large organelles and unbroken cells and the resulting supernatant (S9 fraction) was stored at −80 °C. The overall protein concentration was determined using albumin as the calibration standard and a Pierce BCA Protein Assay (Thermo Fisher Scientific, Inc., Rockford IL).

### Evaluation of *at*RA metabolism via ***in vitro*** Incubations

The metabolism of *at*RA in human fetal livers and the enzymes responsible for *at*RA metabolism were first qualitatively evaluated by standard incubation methods as previously described by us^13,15,33^. The specific metabolites formed from *at*RA by recombinant CYP26A1, CYP26B1 and CYP3A7 in comparison to human fetal liver S9 fractions from representative donors was assessed. 5 pmols of CYP26A1, CYP26B1 and CYP3A7 in 1 mL of 100 mM Potassium phosphate (KPi) buffer (pH 7.4) were incubated for 2 mins (CYP26A1) and 10 mins (CYP26B1 and CYP3A7) with 5 µM *at*RA at 37 °C, respectively. The incubations were quenched with ethyl acetate, metabolites extracted as previously described^15^ and the product formation was then measured by LC-MS/MS as described below. For fetal liver S9 incubations two representative livers were chosen based on the mRNA expression levels of CYP3A7 and CYP26A1. In brief, 0.3 mg S9 protein in 1 mL 100 mM KPi buffer were incubated at 37 °C with 5 µM *at*RA. After a pre-incubation of 5 min the reactions were initiated with the addition of NADPH (1 mM final concentration) and allowed to proceed for 10 min.

The formation of *at*RA metabolites was measured in individual human fetal liver S9 fractions from 27 donors as previously described^33^. All of the incubations were conducted under confirmed linear range of time and protein content. Of the *at*RA metabolites monitored, only 4-OH-RA and 4-oxo-RA were detected in fetal liver S9 fractions and therefore the formation of 4-OH-RA and 4-oxo-RA was quantified in the incubations. To correct the metabolic rates for the sequential formation of 4-oxo-RA from 4-OH-RA, the amount of 4-oxo-RA formed was added to the amount of 4-OH-RA formed for analysis of 4-OH-RA formation rate. For each fetal liver, *at*RA (500 nM) was incubated with 0.1 mg S9 protein/mL of 100 mM KPi buffer (pH 7.4) in 37 °C. After a pre-incubation of 10 min the reactions were initiated with the addition of NADPH (1 mM final concentration) and allowed to proceed for 10 min. The reactions were terminated with 3 mL ethyl acetate, internal standard (*at*RA-d_5_, 50 nM) was added and the metabolites extracted by liquid-liquid extraction. The ethyl acetate layer was collected and dried under a stream of nitrogen, and the dry residue reconstituted in 100 µL acetonitrile for MS/MS analysis.

To determine the relative contributions of CYP26 and CYP3A7 enzymes in *at*RA oxidation in the fetal livers, CYP selective inhibitors were used to inhibit the target enzymes in incubations with S9 fractions from four representative donors. Fluconazole was chosen as the CYP3A7 specific inhibitor as it has been shown to not inhibit CYP26A1^16^. Talarozole was chosen as the CYP26 inhibitor based on previous characterization of its potency towards CYP26A1 and CYP26B1^34^. Ketoconazole was included in the analysis as it is a well characterized pan-CYP inhibitor with high potency both towards CYP3A7 and CYP26s^16,34^. The inhibition of *at*RA metabolism by fluconazole (with CYP3A7) and by talarozole (with CYP26A1 and CYP3A7) was confirmed as previously described^33^. In brief, for fluconazole inhibition, *at*RA (10 µM) was incubated for 10 min with CYP3A7 (5 pmol/mL) in the presence and absence of fluconazole (300 µM) and the percent decrease in 4-OH-RA and 4-oxo-RA formation was quantified. For talarozole inhibition, *at*RA (500 nM) was incubated for 10 mins with CYP3A7 (5 pmol/mL) or 2 mins with CYP26A1 (2 pmol/mL with added 4 pmol/mL P450 reductase) in the presence and absence of talarozole (200 nM). All of the samples were extracted and analyzed by HPLC-MS/MS. Based on the data collected using recombinant enzymes, fluconazole was used at 200 µM to selectively inhibit CYP3A7 and talarozole was used at 200 nM to selectively inhibit CYP26 in human fetal liver S9 fractions from four representative donors. In addition, ketoconazole was tested at 10 µM concentration as a pan-CYP inhibitor but it is also likely more potent inhibitor of CYP3A7 than fluconazole. *at*RA concentration was 500 nM. Incubations were performed as described above for fetal livers and analyzed for metabolite formation by HPLC-MS/MS. The percent inhibition was calculated by comparing the metabolite formation velocity in the presence of the inhibitor to vehicle control.

### LC-MS/MS methods of quantification of retinoid metabolites

The formation of *at*RA metabolites was measured using an Agilent 1290 Infinity UHPLC (Agilent Technologies, Santa Clara, CA) with an Agilent Zorbax C18 column (3.5 µm, 2.1 mm × 100 mm) and coupled to an AB Sciex API 5500 Q/LIT mass spectrometer (AB Sciex, Framingham, MA) as previously reported^15^ with minor modifications on the chromatography^33^. In brief, analytes were separated using a gradient elution as follows: starting from 10:90 acetonitrile: aqueous to 1:1 acetonitrile: aqueous over 0.5 min then increased to 85:15 acetonitrile: aqueous over 4 min and then after a 0.1 min hold increased to 95:5 acetonitrile: aqueous held for 2.5 min. The aqueous phase contained 0.1 % formic acid throughout. Analytes were detected using negative ion electrospray and monitoring MS/MS transitions of *m/z* 299 → 255 Da (*at*RA), *m/z* 315 → 253 Da (4-OH-RA), *m/z* 313 → 269 Da (4-oxo-RA), *m/z* 315 → 241 Da (16-OH-RA) and *m/z* 316 → 272 Da (4-oxo-RA-d_3_). Retinoid concentrations were quantified using Analyst software and peak height ratios between the analyte and the internal standard (4-oxo-RA-d_3_).

### Characterization of CYP3A7 specific activity and Testosterone Metabolism in fetal livers

Testosterone hydroxylation (formation of 6βOH-testosterone) was used as a CYP3A7 specific probe reaction and analyzed as previously described^33^. All incubations were performed in triplicate. Testosterone (at 100 µM, final concentration) was incubated with 0.2 mg hFL S9 protein/mL of 100 mM KPi buffer (pH 7.4). All incubations were performed using 96-well plates with a total volume of 0.1 mL per well. The mixtures were pre-incubated for 10 minutes and reactions were initiated by the addition of NAPDH (1 mM final concentration). After 10 minutes 80 µL of the incubations were added to 80 µL ice-cold acetonitrile to quench the reaction. The samples were then centrifuged at 4 °C for 20 min at 612 g, and an aliquot of the supernatant was collected for LC-MS/MS analysis. 6βOH-testosterone was quantified based on a standard curve of 6βOH-TST (10 to 500 nM). 6βOH-TST was analyzed using a Shimadzu XR DGU-20A5 UFLC (Shimadzu Scientific Instruments, Columbia, MD) coupled to an AB Sciex 3200 Mass Spectrometer (AB Sciex, Framingham, MA). Chromatography was done using a Zorbax SB-C_18_ column (5 µm, 2.1 × 50 mm, Agilent Technologies, Palo Alto, CA) and gradient elution (0.3 mL/min) from initial 5:95 acetonitrile: aqueous 0.1% formic acid held for 2 min and the increased to 100% acetonitrile over 2 min and held for 1.5 min before returning to initial conditions for a re-equilibration period of 3.5 min. 6βOH-TST was detected using a mass transition of *m/z* 305 → 287 Da and positive ion electrospray at source parameters of 5500 V and 450 °C. Metabolite formation was quantified based on peak height and linear standard curve using Analyst software.

### ***In vitro*** to ***in vivo*** Scaling of *at*RA metabolism and Statistical Analysis

Statistical analyses were performed using Prism v.5 (GraphPad Software, Inc., La Jolla, CA). Correlation between metabolite formation velocity from *at*RA and testosterone as substrates in human fetal livers was tested using linear regression. Differences between *at*RA metabolite formation in the presence and absence of inhibitors were tested by one-way analysis of variance. Differences in *at*RA metabolism between fetal livers collected at gestational weeks 10–12, 12–14, 14–16, and 16–20, between different genders of the fetus, and different races were tested by one-way analyses of variance coupled with Bonferroni’s Multiple Comparison Test. A *p* value < 0.05 was considered significant for all statistical analyses.

The overall clearance of *at*RA by fetal liver was predicted using standard *in vitro*-to-*in vivo* scaling methods^35,36^. First, the overall intrinsic clearance (Cl_int_) of *at*RA metabolism in the fetal liver was calculated by summing 4-OH-RA and 4-oxo-RA formation from *at*RA in the fetal liver S9 fraction incubations for each individual donor and the Cl_int_ scaled to the whole liver using Eq. 1 as described previously for adult liver^13^:

1 $$C{l}_{int,FL}=C{l}_{intpermgS9proteinFL}\times \frac{mg\,S9\,protei{n}_{FL}}{{g}_{FL}}\times total\,{g}_{FL}$$

in which FL refers to fetal liver and the total g of fetal liver (liver weight) is specified for the specific gestational ages in Table 1. The hepatic extraction ratio (ER_FL_) by fetal liver for *at*RA was calculated from Eq. 2 based on the well-stirred model of the liver^37,38^:

2 $$E{R}_{FL}=\frac{{f}_{u}\ast C{l}_{int,FL}}{0.5\ast {Q}_{umbilicalvein}+{f}_{u}\ast C{l}_{int,FL}}$$

in which fetal liver blood flow is calculated as 0.5 times the blood flow of the umbilical vein (Q_umbilical vein_) based on the physiology that half of the umbilical vein flow goes to fetal liver and the rest goes to the fetal heart^39^. The Q_umbilical vein_ for each gestational age is listed in Table 1. The plasma unbound fraction of *at*RA (f_u_) used was 0.01 based on previous report^13^.
